# A Novel Study on the Role of Pressure on Surface Adsorption from Solutions

**DOI:** 10.1021/acs.jpcb.3c01492

**Published:** 2023-05-25

**Authors:** N. Sharifi, Tristan Liu, S. M. Clarke

**Affiliations:** Institute for Energy and Environmental Flows and Department of Chemistry, University of Cambridge, Lensfield Rd, Cambridge CB2 1EW, U.K.

## Abstract

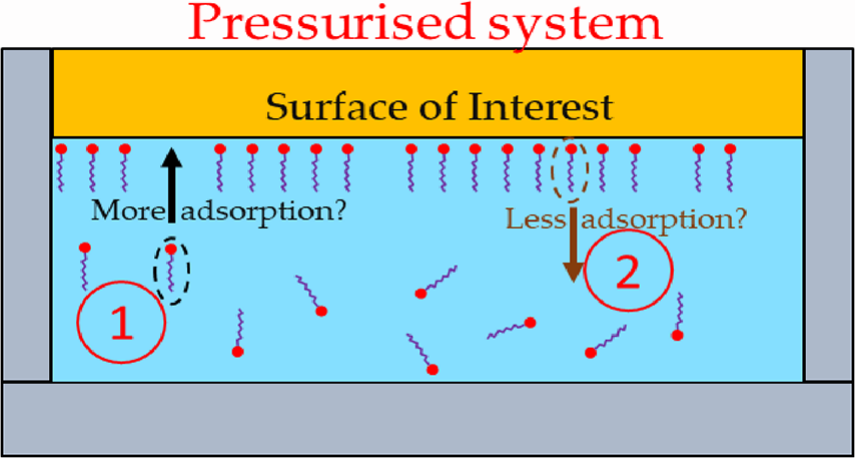

In this work, we present experimental data on the behavior of model additives adsorbed at the solid/liquid interface as a function of pressure. We report that some additives adsorbed from non-aqueous solvents exhibit rather little variation with pressure, while others exhibit more significant changes. We also display the important pressure dependence of added water. This pressure dependence is relevant, indeed central to many commercially important situations where the adsorption of molecular species to the solid/liquid interface under high pressure is key, such as wind turbines, and this work should help in understanding how protective, anti-wear, or friction-reducing agents can persist (or not) under these extreme conditions. With a very significant gap in the fundamental understanding of the role of pressure on adsorption from solution phases, this important fundamental study provides a methodology to investigate the pressure dependence of these academically and commercially important systems. In the best case, one may even be able to predict which additives will lead to more adsorption under pressure and avoid those that may desorb.

## Introduction

In this report, we present a number of experimental methods and results addressing the adsorption of molecular species from non-aqueous solvents to solid surfaces and the variation with concentration and pressure. Although there are many reports of adsorption of additives from water, there is rather less from non-aqueous solutions and almost none on the variation with pressure. This latter point is interesting as many commercial systems where molecular additives that work by adsorption are specifically included are intended to be used under pressure, such as in bearings and lubricated joints of very high (GPa) pressures in modern wind turbines.^1^ Equally more modest loadings and pressure can be found in biomedical systems such as synovial fluids in joints.^2^

However, there is a very significant gap in the fundamental understanding of the role of pressure on adsorption from solution phases. There is very little literature on this topic partly because of the historical challenge of observing molecular layers at all, let alone under extreme conditions. A recent study by Koo et al.^3^ studied the role of pressure on the adsorption of proteins at the aqueous–solid interface and demonstrated that pressure plays an important role in the adsorption amount and correlated the protein affinity for surface adsorption with the bulk Gibbs free energy of unfolding. A similar study carried out by Wirkert et al.^4^ demonstrated the pressure-induced adsorption of lysozyme at a hydrophobic solid substrate, where the layer thickness (monolayer formation) at ambient pressure was reported to grow under elevated pressure, 5 kbar. A pressure study of a non-aqueous system reported by Hirayama et al. reported the “growing” behavior of an adsorbed additive layer onto a metal surface due to high pressure by means of neutron reflectometry in conjunction with cross-sectional imaging by frequency-modulation atomic force microscopy.^5^ There have also been other studies that report pressure dependence in a range of different systems including adsorption of insulin on chromatographic surfaces,^6^ enzymatic activity onto various surfaces,^7^ pressure reversal of anesthesia,^8^ and pressure-induced structural changes of surface-adsorbed polymer films^9,10^ and proteins.^11^ There have also been other studies that report surface-adsorbed layers that are insensitive to pressure.^12^

Therefore, the role of pressure and what determines a system’s sensitivity to pressure are still not very well understood. In this paper, we present a systematic study on the role of pressure on three different systems that provide key insights into the role of pressure on adsorption. We employ solution depletion measurements in a specially designed sample cell to determine the amounts of material adsorbed as a function of pressure. The experimental systems were selected as they have particular relevance/significance in the field of friction and wear, but they are also representative of a much wider class of additives that function by adsorption central to a number of academic and commercial purposes, such as corrosion prevention and wettability control agents.

## Experimental Section

### Materials

Iron(III) oxide powder, 99.999%, (trace metal basis), −100 mesh was obtained from Thermo Scientific and found to have a surface area of 2.9 m^2^ g^–1^ (determined by N_2_ adsorption fitted to the BET adsorption equation). Anhydrous heptane (purity, >99.5%) and cyclohexane (purity, >99.5%), stearic acid (grade I, ≥98.5%), and acetic acid (purity, >99.7%) were all purchased from Sigma. The anhydrous solvents were further dried with molecular sieves for over a week before any measurements were taken. Water-saturated solvents were obtained by equilibrating with ultrapure water over a week before separating the water and the saturated solvent.

### Depletion Isotherms

Solution depletion adsorption isotherms are a useful technique for extracting quantitative adsorption data. A powdered adsorbent is gently mixed with a solution of the adsorbing species of known initial concentration. The concentration of the supernatant is remeasured once equilibrium is established. The fall in the solution concentration is taken to indicate the adsorbed amount. In this study, two different methods/cells were used to study adsorption isotherms, bottle isotherms and a new pressure cell, both of which are now briefly discussed.

### Bottle Isotherms

As described above, 2 g of iron oxide powder is mixed with 20 mL of solution (over a concentration range of 0–15 mM) and placed in a well-sealed glass bottle. The bottle is gently tumbled until equilibrium is reached. The iron oxide powder is then left to sediment, and the supernatant solution is extracted for FTIR analysis.

### Pressure Cell

**Figure 1:**
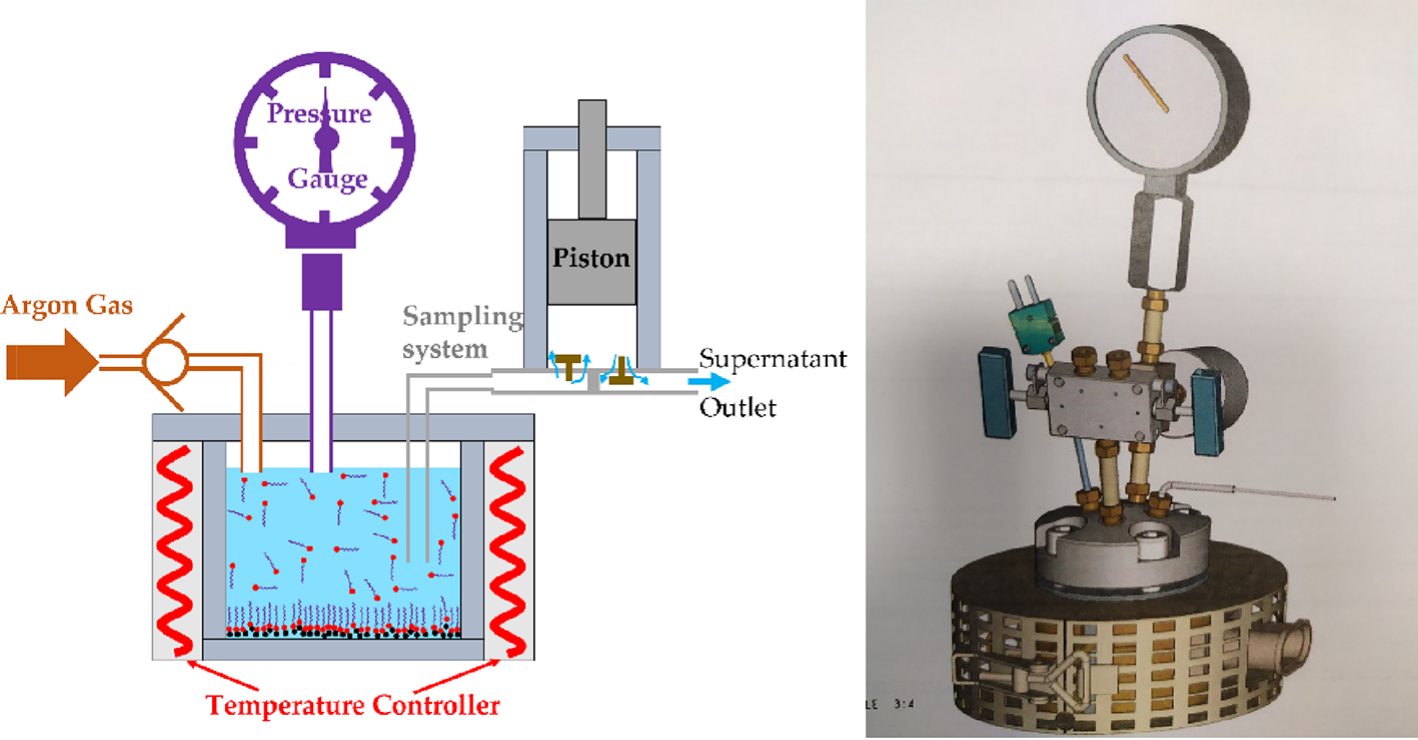
Schematic and image of the high-pressure system. A solution of solvent (blue), additive, and powder (black circles) is mixed continuously. Argon gas is used to pressurize the solution through a one-way valve (brown color region), and the pressure in the cell is recorded through a pressure gauge (purple). A piston sampling system (gray color region) is used to extract the supernatant while the pressure in the cell is maintained at the desired value.

A custom-made cell was purchased from Top Industrie that enables the adsorption isotherms to be measured under pressure. The basic principle of the cell is exactly as that of the bottle isotherms technique: Here, 0.8 g of iron oxide powder is mixed with 8 mL of solution of known concentration in the pressure cell and is mixed using a stirrer bar until equilibrium is reached. The key experimental capability here is that it is possible to control the pressure in the cell and extract the supernatant for FTIR analysis while the sample is under pressure. A schematic and picture of the pressure cell are presented in Figure 1. An argon cylinder is used to pressurize the solution to the desired level, and the sample is then allowed to mix/equilibrate before a piston sampling system is used to extract the supernatant solution while the pressure in the system is maintained at the desired value. A comprehensive background study was carried out to determine the operating procedure that produced consistent and reliable data; this is presented further down.

### FTIR

**Figure 2:**
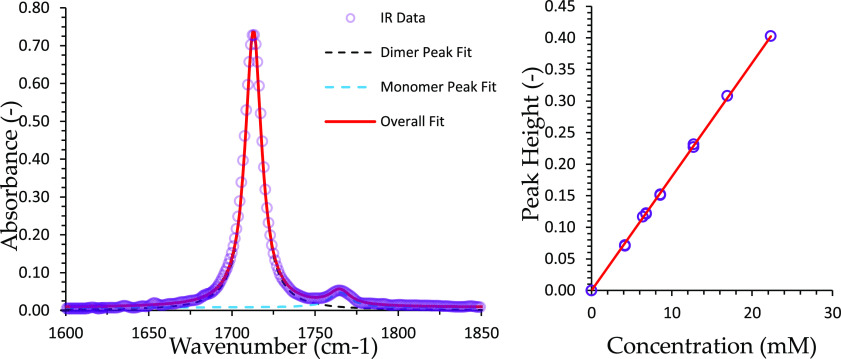
(Left) Example of the FTIR spectrum of stearic acid in cyclohexane (left) showing the monomer (1765 cm^–1^) and dimer peak (1712 cm^–1^). The IR spectrum is fitted to two Lorentzian profiles, and the peak heights are extracted. (Right) Plot of the total peak height (i.e., the sum of the monomer and dimer peak height) against the concentration showing a linear relationship, calibration plot.

The final acid concentration was evaluated by integration of characteristic carbonyl, C=O, peak areas in the IR spectra and comparison to a calibrated set of standard concentrations. The FTIR data were collected using a Bruker Vertex V70 device at the Department of Chemistry, Cambridge, with a sealed liquid cell from Specac with CaF_2_ windows and a path length of 1 mm and 2% resolution, the stated resolution of the device. An example of the FTIR spectral data with the fitted profiles is given in Figure 2 (left). Several samples at different known concentrations are used to give the corresponding calibration plot presented in Figure 2 (right).

## Results

**Figure 3:**
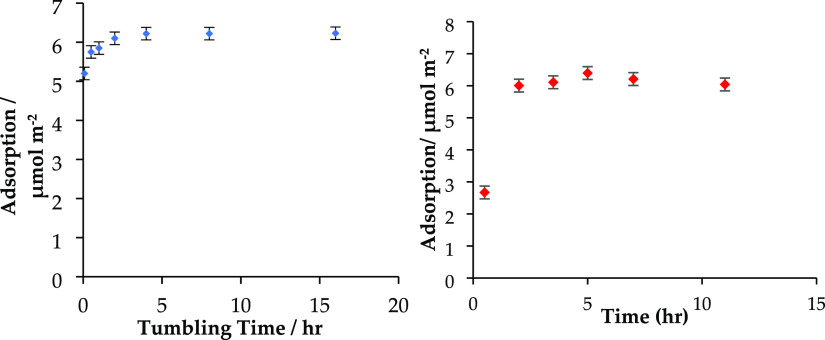
Kinetic study; adsorption isotherm of 15 mM solution as a function of mixing time for bottle isotherm method (left) and pressure cell isotherms (right), showing an equilibration time of 2 h (20C).

Kinetic and method validation studies were carried out to determine the time required to reach equilibrium both in the bottle measurements and in the pressure cell. The kinetic measurements were performed by measuring the adsorbed amount of an initially 15 mM solution where the mixing/equilibration step is performed for different lengths of time. The results of the kinetic studies are presented in Figure 3. This figure shows that for the shortest times investigated, the solutions were still equilibrating, but the measured concentrations do quickly equilibrate to a plateau value. The equilibrium times for bottle isotherms (mixed by tumbling) are approximately 4 and 2 h for the high-pressure cell method (mixed by stirrer bar in the device). Subsequently, all isotherms were equilibrated for 10 h in the bottle isotherms and 4 h in the high-pressure cell to be confident of achieving equilibrium.

**Figure 4:**
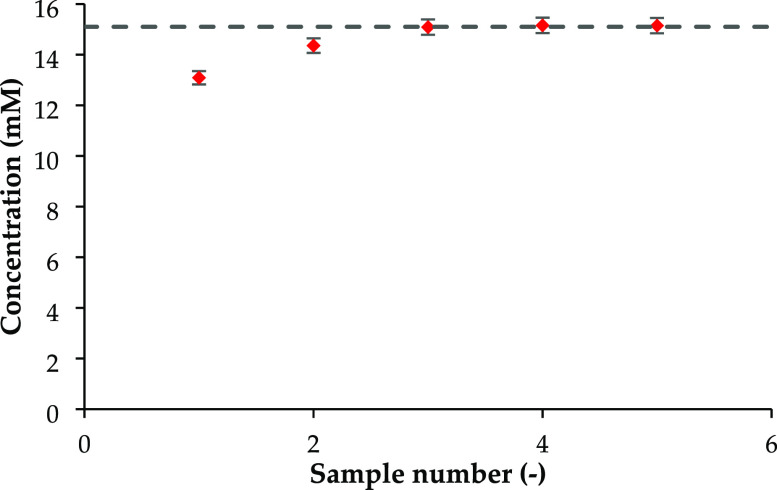
The concentration of samples extracted shows a plateau after the third sample. The final three samples are at the same concentration as the input solution (dashed line), suggesting that the tubes in the sampling system are saturated and do not contribute to further adsorption of the stearic acid from the supernatant solution.

In measuring the adsorption isotherms within the pressure cell, it is important to ensure that only the adsorption of the additive on the iron oxide is measured. Although the inside of the cell is made of Teflon, the tubes within the sampling system used to extract the supernatant solution are made of metal (316L stainless steel), which may provide additional adsorption sites for the additive. To deconvolute this additional adsorption from the adsorption on the iron oxide powder, pure solutions of stearic acid in cyclohexane (15 mM) were placed within the cell, and subsequently, five samples, 1 mL each, were extracted one at a time and the concentrations were measured (Figure 4). The concentration from the first extraction is lower than that of the solution put into the cell due to adsorption onto the tubes of the sampling system. The concentration of the second extraction is much closer to the cell concentration, indicating less adsorption onto the tubes. The third, fourth, and fifth extractions all have the same concentration as the input solution. Therefore, the adsorption of stearic acid within the pressure cell tubes is completely saturated after the third extraction. For all experiments presented here, five samples were extracted and only the fourth and fifth extracts were used to measure the supernatant concentration.

### Temperature Study

**Figure 5:**
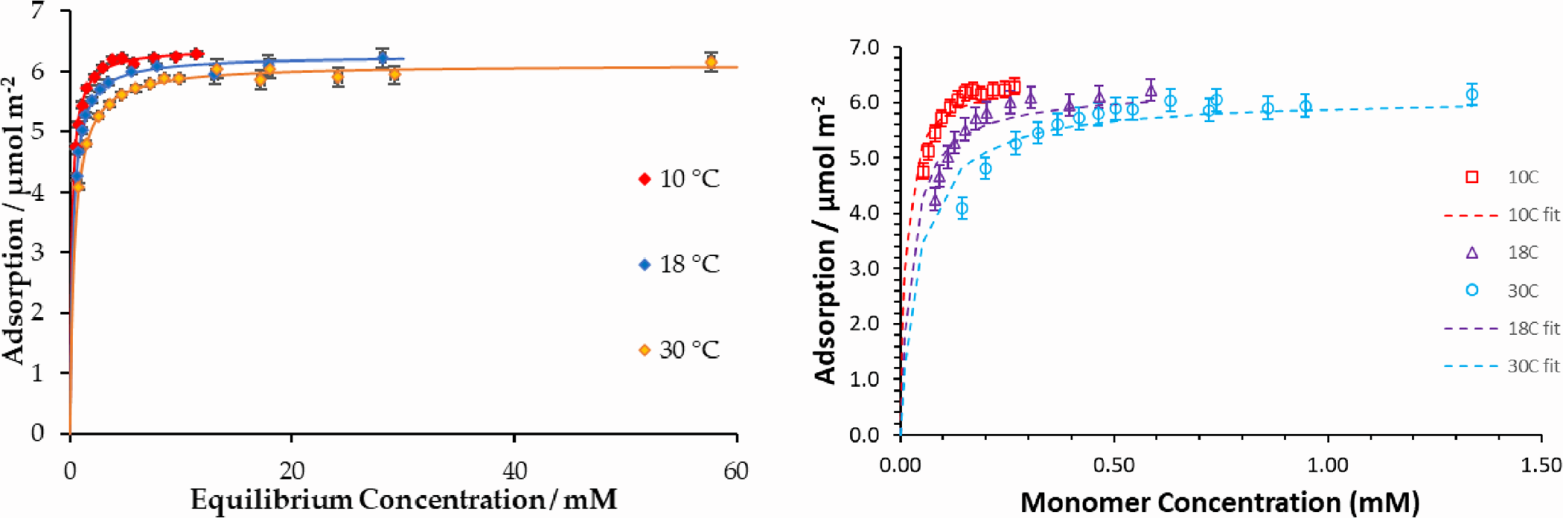
(Left) Plot of adsorption isotherms of stearic acid from cyclohexane at 10 °C (red), 18 °C (blue), and 30 °C (orange) and the corresponding fitted Langmuir models. (Right) Adsorption isotherm plotted as a function of the monomer concentration.

Initially, the bottle isotherm method was used to measure the adsorption isotherm of stearic acid in cyclohexane onto iron oxide at different temperatures (Figure 5). The adsorption isotherm shows the expected rise from low concentrations to a plateau and follows a Langmuir isotherm model reasonably well:
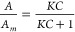
1where *A* is the adsorption amount, *A_m_* is the maximum adsorption at high concentration, *K* is the Langmuir constant, and *C* is the equilibrium concentration.

However, in calculating the thermodynamic variables of adsorption, it is important to consider the role of dimerization of the fatty acids, characterized by a thermodynamic association (dissociation) constant. Based on the reported values of these constants,^13^ it is possible to calculate the extent of dimerization of the stearic acid in cyclohexane. For example, at 15 mM and 25C, approximately 95% of the acids exist as a dimer. Therefore, self-association-corrected adsorption *K* values are also presented here, as described in the SI.

The experimental data and analysis above enable the thermodynamic adsorption parameters to be extracted, as summarized in Table 1. The plateau value corresponds to an area per molecule of 26.5 Å^2^, which is similar at all temperatures. This value suggests a well-packed complete monolayer of upright molecules. The temperature dependence of the thermodynamic constants allows the enthalpy and entropy to be calculated, as shown in Table 2.

**Table 1:** Summary of the Langmuir Model Parameters (Maximum Adsorbed Amount, *A_m_*; Langmuir Constant, *K*; and Association Constant of Stearic Acid, *K*_association_) Fitted to the Adsorption Isotherms

	temperature (°C)		
adsorption parameter	10	18	30
*A_m_* (μmol m^–2^)	6.39 ± 0.02	6.26 ± 0.04	6.10 ± 0.04
*K* (M^–1^)	(5.67 ± 0.17) × 10^3^	(3.61 ± 0.13) × 10^3^	(2.49 ± 0.23) × 10^3^
*K*_association_ (mM^–1^)	78.1	40.2	15.7
dimerization-corrected *K* (M^–1^)	8.84 × 10^4^	4.21 × 10^4^	2.61 × 10^4^

**Table 2:** Thermodynamic Parameters for the Adsorption of Stearic Acid onto Iron Oxide from Cyclohexane

Δ*H*_adsorption_ (kJ mol^–1^)	–28.9 ± 4.3
Δ*S*_adsorption_ (J K^–1^ mol^–1^)	–31 ± 15

### Pressure Cell

**Figure 6:**
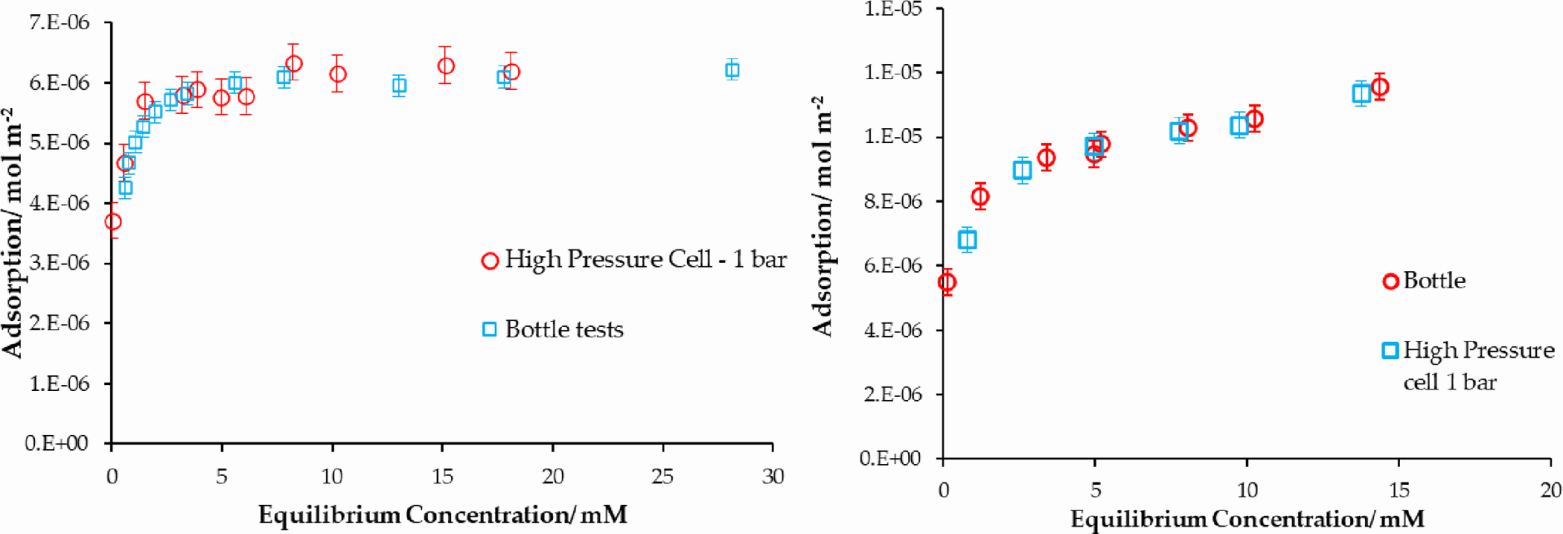
Adsorption isotherms measured in the bottle method compared to the high-pressure cell at 1 atm. (Left) Stearic acid from cyclohexane onto iron oxide. (Right) Acetic acid from heptane onto iron oxide.

Adsorption isotherms of stearic acid from cyclohexane and acetic acid from heptane onto iron oxide were also measured using the high-pressure cell at ambient temperature (20C) and pressure (1 atm). The data obtained from the same system in the bottle isotherm and pressure cell at 1 atm are presented in Figure 6. The agreement is good, particularly given the experimental challenges of using the pressure cell. Therefore, we can be confident in the operating method used for the pressure cell.

### Pressure Dependence

The role of pressure on adsorption from solution was studied for both systems: (a) stearic acid/cyclohexane and (b) acetic acid/heptane. Different solvents were used for their different bulk compressibilities, as discussed further below.

**Figure 7:**
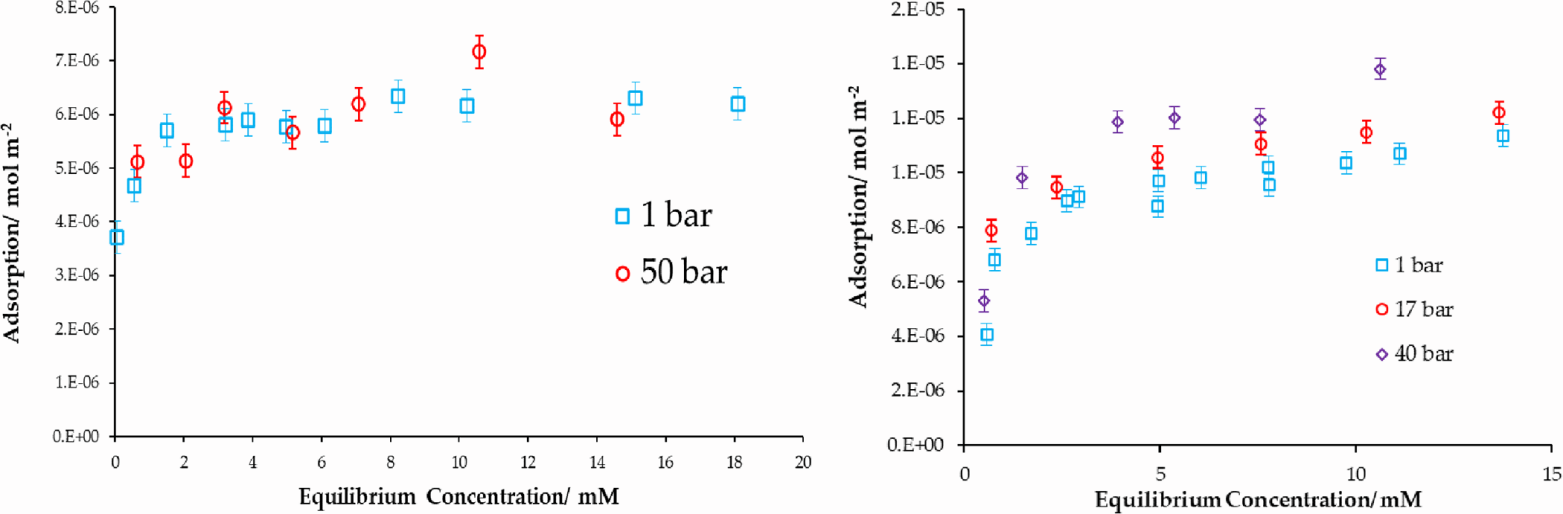
(Left) Adsorption isotherms of stearic acid in cyclohexane at 1 atm and 50 bar with no significant changes with pressure. (Right) Adsorption isotherms of acetic acid from heptane at three different pressures.

In the case of stearic acid in cyclohexane, the adsorption isotherms between 1 atm and 50 bar do not show any significant changes (Figure 7).

However, the adsorption isotherms of acetic acid adsorbed from heptane at three different pressures show a clear and systematic increase in the amount adsorbed with increasing pressure. Adsorption increases with pressure over the whole profile of the isotherm, including at high concentrations in the plateau region. The adsorption isotherms are fitted to Langmuir models, and the Langmuir model parameters are presented in Table 3.

**Table 3:** Summary of the Langmuir Model Parameters Fitted to the Adsorption Isotherms as a Function of Pressure^a^

	pressure (bar)		
adsorption parameter	1	17	40
*A_m_* (μmol m^–2^)	10.8 ± 1	12.0 ± 1	13.5 ± 1
*K* (M^–1^)	(2.0 ± 0.3) × 10^3^	(1.8 ± 0.3) × 10^3^	(1.5 ± 0.3) × 10^3^
dimerization-corrected *K* (M^–1^)	(1.7 ± 0.3) × 10^3^	(1.6 ± 0.5) × 10^3^	(1.4 ± 0.5) × 10^3^

a This is for the adsorption of acetic acid on iron oxide in heptane. The association constant used to calculate the acetic acid monomer concentration at 25C is 3.1 × 10^–4^ mM as reported in the literature.^17^

The adsorbed amount increases with increasing pressure for acetic acid, suggesting an increase in the packing density of the adsorbed layers at the surface. The bulk isothermal compressibility coefficient, κ_p_, of acetic acid is calculated to be 8.4 × 10^–5^ bar^–1^. Similarly, the surface density data from Table 3 enables us to estimate the surface-adsorbed layer compressibility coefficient, κ_p_, to be 6.1 × 10^–3^ bar^–1^. The compressibility ratio of acetic acid in the bulk and surface is significantly different with the 2D layer being approximately 2 orders of magnitude bigger than the bulk. There is evidence that these 2D layers are more susceptible to expanding/contracting relative to 3D crystals partly due to a change in the coordination number.^14,15^ This is also supported by data reported in the literature, where the pure stearic acid bulk isothermal compressibility coefficient is approximately 7.9 × 10^–5^ bar^–1^ and the 2D compressibility coefficient of stearic acid Langmuir layer at the air–water interface in the condensed phase^16^ is 3.0 × 10^5^ bar^–1^; the 2D layer is 10 orders of magnitude more compressible. (Details of the compressibility calculation are provided in the Supporting Information).

### Multicomponent Systems: The Role of Water

Water is important in controlling behavior in both the bulk and at the surface. In the bulk, where acids dimerize,^18,19^ water can also hydrogen bond with the acid. This changes the acid monomer concentration in the bulk, which in turn will change chemical potential, which in turn controls adsorption. Water can also directly compete for adsorption sites.

The adsorption isotherm of stearic acid at different water concentrations was studied through bottle solution depletion isotherms at atmospheric pressure. In the first experiment, (a) dry cyclohexane was obtained by drying with molecular sieves over a week. (b) The water-saturated cyclohexane was obtained by mixing cyclohexane and water for over a week and removing the saturated cyclohexane from the top of the bottle. The water content of this saturated cyclohexane is independently measured to be 2.3 mM through Karl Fischer titration. (c) In the third case, 1% of water was added to the cyclohexane (corresponding to 400 mM). However, it is important to note that this 1% is well above the solubility limit, and the water visibly phase separates into small droplets. The higher concentration of water was used to ensure that the solvent remains water-saturated, even if water is significantly adsorbed. It is expected that the chemical potential of water will not change significantly above the solubility limit (2.3 mM).

While water does phase separate, the solution is thoroughly mixed to prevent mass transfer problems. It is important to note that the physical volume of the water is very small compared to the organic solvent phase and the iron oxide powder, and the 1 wt % is not enough to contain all the iron oxide powder in the water phase and stop the iron oxide from being in contact with the organic phase. The aim of this experiment is to investigate if water can outcompete stearic acid on the iron oxide surface at the highest concentration.

**Figure 8:**
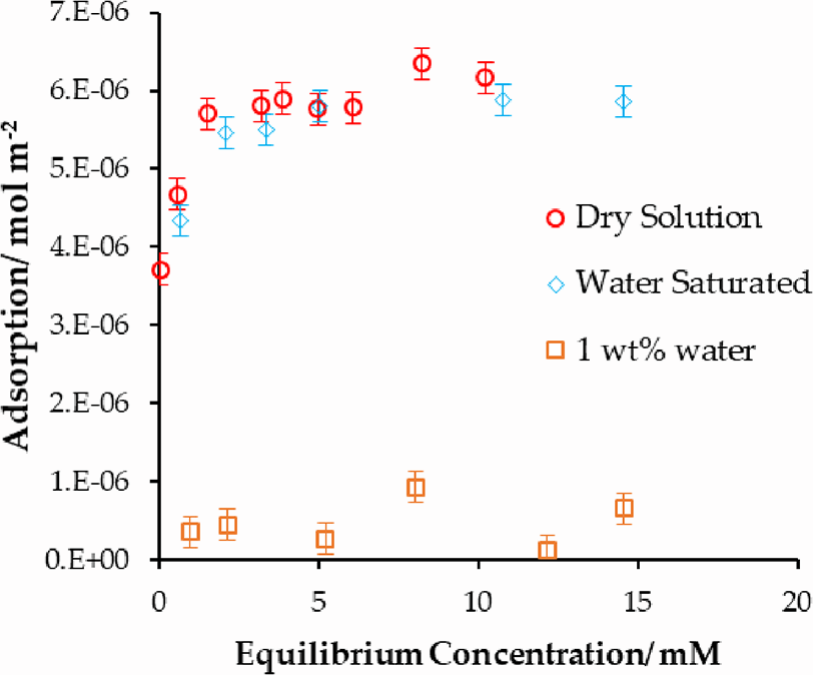
Adsorption isotherm of stearic acid onto iron oxide from cyclohexane at different water concentrations. This result demonstrates that water can outcompete and displace stearic acid onto the surface of iron oxide.

The adsorption isotherms of stearic acid at different water concentrations are shown in Figure 8. This figure shows that an initially water-saturated cyclohexane results in a small reduction in the adsorption of stearic acid. This is attributed to some amount of water adsorption reducing the amount of water present below the saturated level, which minimizes further water adsorption.

However, in the presence of 1 wt % water (400 mM), the adsorption of stearic acid reduces very significantly to less than 10% of the adsorbed amount in the dry solution. This clearly suggests that water can outcompete the stearic acid and preferentially adsorbed onto the surface but requires “free” water to do so (or at least a completely saturated solvent). This is an important result as this shows that excess water present in the system can essentially completely remove the additive from the surface.

### Pressure Dependence with Water

**Figure 9:**
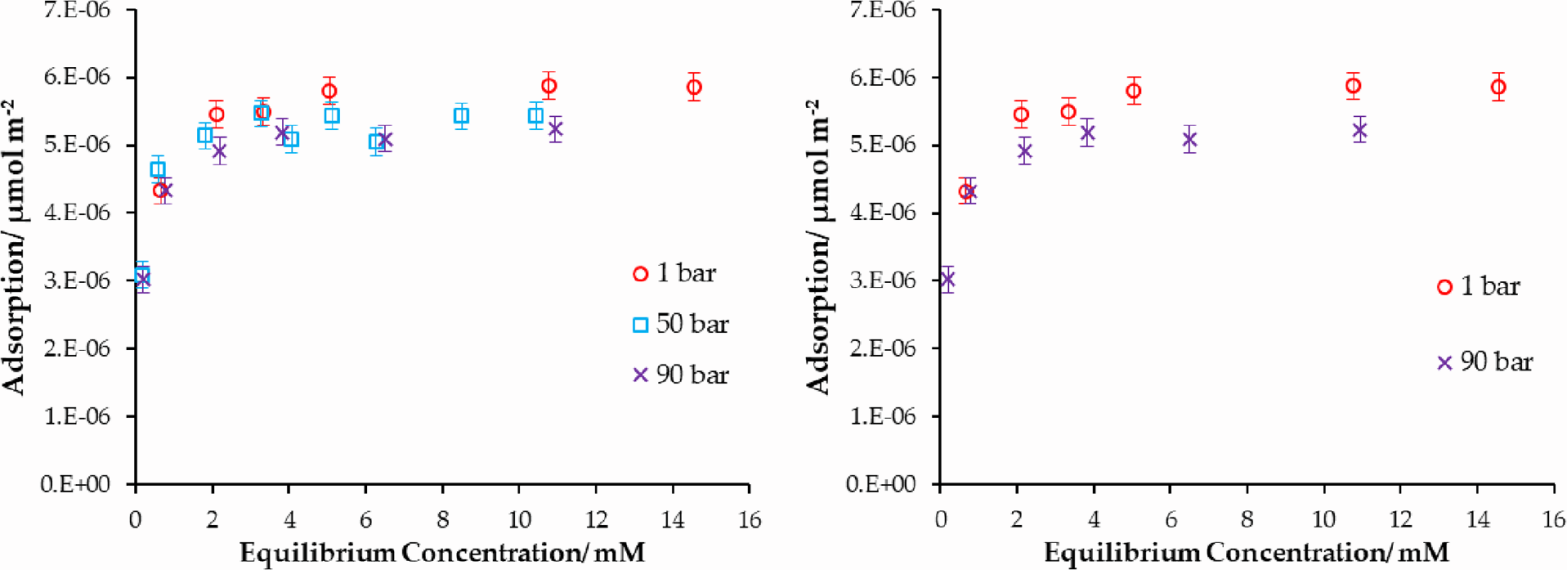
(Left) Adsorption isotherm of stearic acid onto iron oxide from water-saturated cyclohexane (water concentration of 2.3 mM). Increasing the pressure decreases the amount of stearic acid adsorbed onto the surface due to the increased adsorption of water onto the surface. (Right) Same plot with 50 bar data removed for clarity.

In the case of stearic acid adsorption from anhydrous cyclohexane, the system showed no significant changes with pressure (Figure 7 above). The pressure dependence of the same system is investigated again, with the cyclohexane saturated with water (2.3 mM initial water concentration) and the adsorption isotherms presented in Figure 9. While there is some scatter in the low-concentration region, however, comparing the 1 bar and 90 bar data, there is a modest fall in the adsorbed amount of stearic acid as a function of pressure.

As demonstrated in Figure 8, water can outcompete stearic acid onto iron oxide. The results in Figure 9 suggest that pressure may be able to amplify this effect further. The results are summarized in Table 4 where the adsorption isotherm Langmuir constant and plateau values are presented.

**Table 4:** Summary of the Langmuir Model Parameters Fitted to the Adsorption Isotherms as a Function of Pressure^a^

	dry solution pressure (bar)		wet solution pressure (bar)		
adsorption parameter	1	50	1	50	90
*A_m_* (μmol m^–2^)	6.26 ± 0.1	6.26 ± 0.1	5.92 ± 0.1	5.45 ± 0.3	5.3 ± 0.2
*K* (M^–1^)	(3.61 ± 0.1) × 10^3^	(3.61 ± 0.2) × 10^3^	(4.3 ± 0.2) × 10^3^	(7.9 ± 0.4) × 10^3^	(6.5 ± 0.3) × 10^3^

a This is for the adsorption of stearic acid on iron oxide in dry and wet cyclohexane.

## Discussion and Conclusions

The overall goal of this area of research is to determine the pressure-dependent adsorption of additives used commercially. The concern at present is that formulation work under ambient pressure is not representative of the adsorption under high pressure. Molecular additives that function by adsorption include corrosion inhibitors, friction modifiers, and wetting agents. A formulation that functions well under ambient conditions may fail completely if the additive unexpectedly desorbs under pressure. Alternatively, a pressure-induced increase in adsorption might be exploited such that less additive will be required to achieve the commercial function under pressure. These changes will become increasingly significant under very high pressure. This is particularly relevant to wind turbines, for example, which have pressures of the order 3 GPa.

We have presented some initial pressure-dependent isotherm measurements, which indicate that there are changes in adsorption even for relatively modest pressure increases (up to 50 atm). These results strongly suggest that one would expect much more significant changes when the imposed pressure is some 1000 times greater.

However, the experimental adsorption measurements are technically challenging, even under the rather modest pressures investigated here. Hence, it would be more convenient if we could use relatively straightforward adsorption or other measurements under ambient conditions that could be used to reliably predict the adsorption. Here, we consider a number of possible approaches to address this and hope that this will prompt further theoretical work.

**Figure 10:**
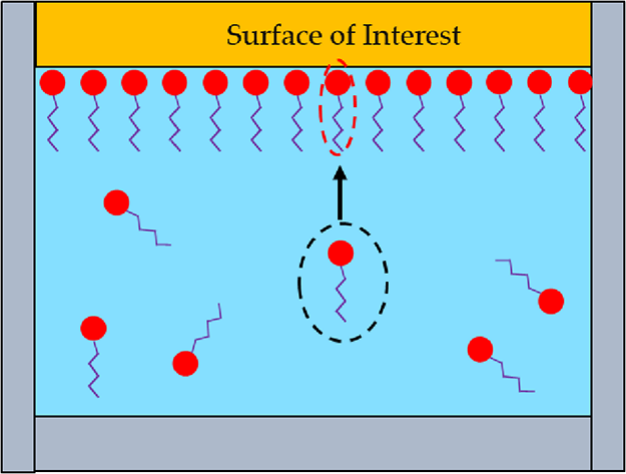
Illustration of a dense well-packed monolayer adsorbed onto the surface. The dashed circles around the molecules represent the apparent volume occupied by the additive molecule. The volume of the additive molecule in bulk solution (black dashed circle) is larger than the volume it occupies on the surface (red dashed circle). In this case, adsorption from the bulk solution to the surface reduces the total system volume; therefore, increasing the pressure would result in increased adsorption.

The thermodynamics of the problem indicates that the pressure dependence of the adsorption will be determined by the changes in the volume of the system on adsorption, as illustrated in Figure 10. The variation, if any, will depend if this change is positive, negative, or zero and will include the net change in volume of the bulk solution and at the surface. While the volume net change at the surface is experimentally very challenging, the change in volume in bulk solution (partial molar volume of expansion/mixing) is significantly easier to experimentally measure.

Concerning the adsorption, it has been found that some alkylated molecular additives form dense well-packed monolayers when adsorbed from the solution (as is the case for stearic acid and acetic acid adsorbed in iron oxide presented here). These layers might be considered to be similar to a “slice” of the bulk additive crystal structure.^20^ Hence, the volume changes of the bulk crystal additive dissolving in the solvent might also provide an estimate of the volume change of the additive adsorption/desorbing from a surface.

In summary, the volume change on adsorption may be similar to that of bulk dissolution. We can measure the volume change of bulk dissolution relatively easily and hence have a very simple predictor of how the adsorbed layer may respond to pressure.

**Figure 11:**
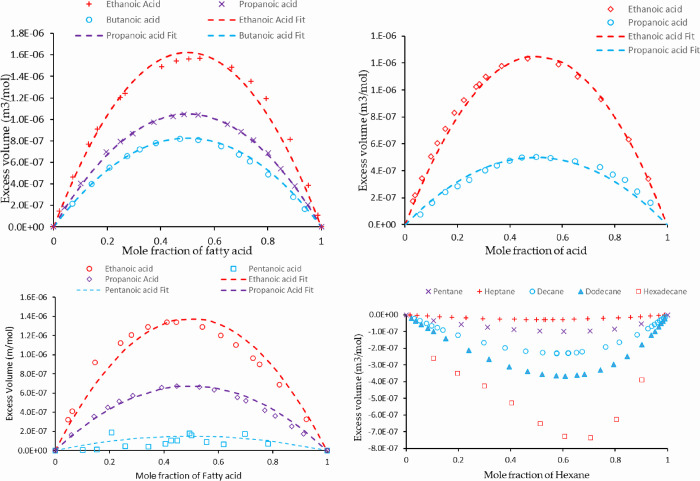
Excess volume of various fatty acids in different solvents. Top left: cyclohexane. Top right: hexane. Bottom left: heptane. Bottom right: mixing of different alkanes in hexane, showing an increasing amount of contraction in the volume as the chain length increases. All data presented are obtained from the literature and are measured using a U-tube densitometer^21−29^ and the corresponding fits. In all solvents, the volume expansion decreases with fatty acid chain length.

The partial molar volume changes on mixing for a number of bulk systems are presented in Figure 11.^21−29^ At all acid concentrations, for all systems presented, the excess volume is positive, i.e., the systems expand on mixing. Interestingly, this excess volume decreases with increasing fatty acid chain length. The volume expansion on mixing is attributed to non-ideal interactions, which could arise due to a combination of both molecular structure and chemical functionality. A significant difference in structures of molecules can result in better or worse packing, therefore resulting in a contraction or expansion of the volume. A difference in the chemical functionality of the molecules, i.e., a polar and non-polar molecule usually, has a very unfavorable interaction and will be expected to give rise to a volume expansion.^30−32^ For the systems studied here, the hydrocarbon chains of the fatty acids are all linear similar to the solvent, heptane. Therefore, it may be reasonable to assume that the expansion volume presented in Figure 11 is mainly due to the polar head group of the acid.

It can be shown that the excess volume on mixing may be modeled most simply by
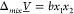
2where *x_i_* is the mole fraction of species *i*. The parameter *b*, which characterizes the volume changes on mixing (), can be used to compare the relative expansion of each system in both the bulk solution and on the surface.

**Figure 12:**
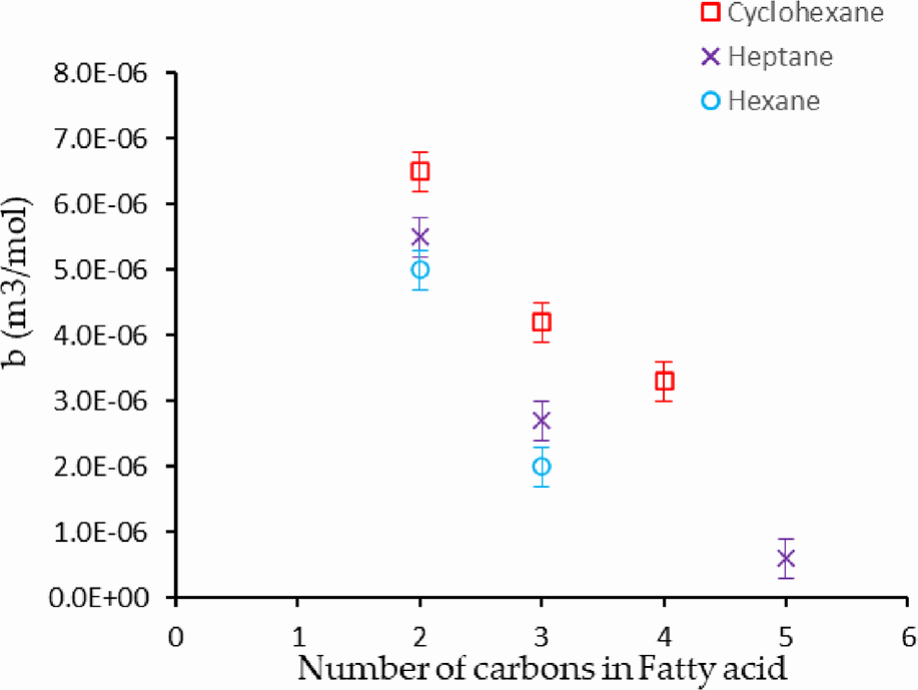
Coefficient *b* obtained from fitting the excess volume of fatty acids in three different solvents; the *b* coefficient is plotted as a function of fatty acid chain length (number of carbon atoms in the acid). The *b* coefficient and hence the excess volume decreases with chain length, as expected, due to a decrease in the average polarity of the fatty acids.

A plot of the parameter *b* as a function of fatty acid chain length is presented in Figure 12, which shows an important trend in all three solvents; the value of *b* decreases with chain length, i.e., the volume expansion on mixing is decreasing with chain length. Therefore, while it is a very long extrapolation, it is not unreasonable to expect stearic acid in cyclohexane to have a significantly smaller or even negative expansion (on dissolution) than acetic acid in heptane. This again supports the idea that the difference arising from the acid head group gives rise to the volume changes, and for larger molecules, this becomes a smaller contribution.

For acetic acid in heptane, the bulk excess partial molar volume of the solution is positive over the concentration region studied here. Therefore, one would expect an increase in the amount adsorbed as a function of increasing pressure for the case of acetic acid in heptane, as observed.

The variation evident in Figure 12 suggests that the parameter *b* for stearic acid in cyclohexane is very small or even negative. The stearic acid expansion is expected to be significantly smaller than the acetic acid in cyclohexane or heptane. In the case of acetic acid in heptane, the adsorption increases with the pressure as expected. However, there are no similar changes in adsorption with pressure for stearic acid in cyclohexane.

In the case of water-saturated cyclohexane, the adsorption of stearic acid decreases with pressure because the water preferentially displaces stearic acid and this effect is further pronounced with increasing pressure. This is tentatively assigned to the water having a much higher polarity than stearic acid, therefore a larger expansion volume on mixing with oil than stearic acid in oil.

## Supporting Information Available

Role of pressure on self-association and bulk compressibility data and model based on literature data (PDF)

## References

[ref1] Brand R. A. Joint contact stress: a reasonable surrogate for biological processes?. Iowa Orthop J. 2005, 25, 82–94. 16089079 PMC1888787

[ref2] Zhai Y.; Chong P. L.-G.; Taylor L. J.-A.; Erlkamp M.; Grobelny S.; Czeslik C.; Watkins E.; Winter R. Physical Properties of Archaeal Tetraether Lipid Membranes As Revealed by Differential Scanning and Pressure Perturbation Calorimetry, Molecular Acoustics, and Neutron Reflectometry: Effects of Pressure and Cell Growth Temperature. Langmuir 2012, 28, 5211–5217. 10.1021/la300142r. 22352806

[ref3] Koo J.; Erlkamp M.; Grobelny S.; Steitz R.; Czeslik C. Pressure-Induced Protein Adsorption at Aqueous–Solid Interfaces. Langmuir 2013, 29, 8025–8030. 10.1021/la401296f. 23725210

[ref4] Wirkert F. J.; Paulus M.; Nase J.; Möller J.; Kujawski S.; Sternemann C.; Tolan M. X-ray reflectivity measurements of liquid/solid interfaces under high hydrostatic pressure conditions. J. Synchrotron Radiat. 2014, 21, 76–81. 10.1107/S1600577513021516. 24365919

[ref5] Hirayama T.; Maeda M.; Sasaki Y.; Matsuoka T.; Komiya H.; Hino M. Growth of adsorbed additive layer for further friction reduction. Lubr. Sci. 2019, 31, 171–178. 10.1002/ls.1420.

[ref6] Szabelski P.; Liu X.; Guiochon G. Pressure-induced effects in the heterogeneous adsorption of insulin on chromatographic surfaces. J. Chromatogr A. 2003, 1015, 43–52. 10.1016/s0021-9673(03)01286-x. 14570318

[ref7] Czeslik C.; Luong T. Q.; Winter R. Enzymatic activity under pressure. MRS Bull. 2017, 42, 738–742. 10.1557/mrs.2017.211.

[ref8] Lever M. J.; Miller K. W.; Paton W. D. M.; Smith E. B. Pressure Reversal of Anaesthesia. Nature 1971, 231, 368–371. 10.1038/231368a0. 4931002

[ref9] Micciulla S.; Gutfreund P.; Kanduč M.; Chiappisi L. Pressure-Induced Phase Transitions of Nonionic Polymer Brushes. Macromolecules 2023, 56, 1177–1188. 10.1021/acs.macromol.2c01979.

[ref10] Reinhardt M.; Dzubiella J.; Trapp M.; Gutfreund P.; Kreuzer M.; Gröschel A. H.; Müller A. H. E.; Ballauff M.; Steitz R. Fine-Tuning the Structure of Stimuli-Responsive Polymer Films by Hydrostatic Pressure and Temperature. Macromolecules 2013, 46, 6541–6547. 10.1021/ma400962p.

[ref11] Jeworrek C.; Steitz R.; Czeslik C.; Winter R. High pressure cell for neutron reflectivity measurements up to 2500 bar. Rev. Sci. Instrum. 2011, 82, 025106 10.1063/1.3553392. 21361632

[ref12] Mia S.; Mizukami S.; Fukuda R.; Morita S.; Ohno N. High-pressure behavior and tribological properties of wind turbine gear oil. J. Mech. Sci. Technol. 2010, 24, 111–114. 10.1007/s12206-009-1179-5.

[ref13] Jaishankar A.; Jusufi A.; Vreeland J. L.; Deighton S.; Pellettiere J.; Schilowitz A. M. Adsorption of Stearic Acid at the Iron Oxide/Oil Interface: Theory, Experiments, and Modeling. Langmuir 2019, 35, 2033–2046. 10.1021/acs.langmuir.8b03132. 30624939

[ref14] Sun C.; Brewer A.; Clarke S. M.; Bhinde T.; Parker J. E. Adsorption of iodoalkanes on graphite. Mol. Phys. 2013, 111, 1005–1014. 10.1080/00268976.2012.762127.

[ref15] Sacchi M.; Brewer A. Y.; Jenkins S. J.; Parker J. E.; Friščić T.; Clarke S. M. Combined Diffraction and Density Functional Theory Calculations of Halogen-Bonded Cocrystal Monolayers. Langmuir 2013, 29, 14903–14911. 10.1021/la402910a. 24215390 PMC3968856

[ref16] El-hefian E. Surface investigation of chitosan film with fatty acid monolayers. Maejo Int. J. Sci. Technol. 2009, 3, 277–286.

[ref17] Fujii Y.; Yamada H.; Mizuta M. Self-association of acetic acid in some organic solvents. J. Phys. Chem. 1988, 92, 6768–6772. 10.1021/j100334a054.

[ref18] Dunken H.; Rudakoff G. Über Die Assoziation Fettsaurer Salze in Unpolaren Lösungsmitteln. Z. Phys. Chem 1962, 219O, 36–46. 10.1515/zpch-1962-21906.

[ref19] Harris J. T. Jr.; Hobbs M. E. A Study of the Association of Some Organic Acids by Infrared Absorption Measurements1. J. Am. Chem. Soc. 1954, 76, 1419–1422. 10.1021/ja01634a084.

[ref20] Von Sydow E. On the Structure of the Crystal Form B of Stearic Acid. Acta Cryst. 1955, 8, 557–560. 10.1107/S0365110X55001746.

[ref21] Lark B. S.; Palta R. C. Excess volumes of acetic acid + benzene, + toluene, + tetrachloromethane, + cyclohexane, and + methanol. J. Chem. Thermodyn. 1980, 12, 101–103. 10.1016/0021-9614(80)90121-4.

[ref22] Yamamoto H.; Sakamoto K.; Bando Y.; Matsumoto S.; Shibata J. Density and Excess Molar Volume of Tri-n-octylamine + Propionic Acid + Diluent at 298.15 K. J. Chem. Eng. Data 1997, 42, 238–242. 10.1021/je9601671.

[ref23] Lark B. S.; Palta R. C. Excess volumes of propionic acid + cyclohexane, +benzene, +tetrachloromethane, +toluene, and +methanol. J. Chem. Thermodyn. 1980, 12, 287–290. 10.1016/0021-9614(80)90048-8.

[ref24] Wright E. H. M.; Akhtar B. A. Equilibrium studies of soluble films of short chain monocarboxylic fatty acids at the organoliquid-vapour phase boundary. Trans. Faraday Soc. 1970, 66, 990–1003. 10.1039/tf9706600990.

[ref25] Yanes C.; Pérez-Tejeda P.; Maestre A. Excess molar volumes and excess refractive indices of (cis-9-octadecenoic acid + n-hexane or cyclohexane or benzene or trichloroethene or tetrachloroethene) at 298.15 K. J. Chem. Thermodyn. 1989, 21, 1217–1222. 10.1016/0021-9614(89)90109-2.

[ref26] Drábek O.; Cibulka I. Excess Molar Volumes of Binary Mixtures of Acetic Acid and Propionic Acid with some Members of Homologous Series of Alkanes. Collect. Czech. Chem. Commun. 1991, 56, 736–744. 10.1135/cccc19910736.

[ref27] Lark B. S.; Banipal T. S. Excess enthalpies and excess volumes of some carboxylic acid mixtures with n-heptane. Thermochim. Acta 1985, 91, 141–149. 10.1016/0040-6031(85)85210-2.

[ref28] Vilcu R.; Lucinescu E.; Dragut M. Excess values in binary systems of carboxylic acids with n-heptane and benzene. Rev. Roum. Chim. 1985, 30, 289–295.

[ref29] Lodl S. J.; Scheller W. A. Isothermal Vapor-Liquid Equilibrium Data for the System n-Heptane - n-Valeric Acid at 50, 75 and 100 °C. J. Chem. Eng. Data 1967, 12, 485–488. 10.1021/je60035a006.

[ref30] Suzuki K.; Taniguchi Y.; Watanabe T. Effect of pressure on the dimerization of carboxylic acids in aqueous solution. J. Phys. Chem. 1973, 77, 1918–1922. 10.1021/j100634a021.

[ref31] Spivey J. P.; Valkó P. P.; William D. M. Applications of the Coefficient of Isothermal Compressibility to Various Reservoir Situations With New Correlations for Each Situation. SPE Reservoir Eval. Eng. 2007, 10, 43–49. 10.2118/96415-PA.

[ref32] Vong W.-T.; Tsai F.-N. Densities, Molar Volumes, Thermal Expansion Coefficients, and Isothermal Compressibilities of Organic Acids from 293.15 K to 323.15 K and at Pressures up to 25 MPa. J. Chem. Eng. Data 1997, 42, 1116–1120. 10.1021/je970005k.

