# Supplementary material for: A Novel Study on the Role of Pressure on Surface Adsorption from Solutions
Source: J Phys Chem B. 2023 May 25;127(22):5141–9. doi: 10.1021/acs.jpcb.3c01492 (PMC10258792; doi:10.1021/acs.jpcb.3c01492)
Supplement: Supplementary file 1 — jp3c01492_si_001.pdf [file jp3c01492_si_001.pdf]

A Novel Study on the Role of Pressure on Surface Adsorption from Solution

N. Sharifi, Tristan Liu, S. M. Clarke*
Institute for Energy and Environmental Flows and Department of Chemistry, University of Cambridge, Lensfield Rd, Cambridge, CB2 1EW, UK

Address correspondence to this author: sc10015@cam.ac.uk

Supplementary information

Role of Pressure on Solution Association:
The association constant of fatty acids dimerization can be pressure dependent. Work by Suzuki et al[32] reports that the association constant of acetic acid in aqueous systems increases with pressure over the pressure range of 0.2-0.3 GPa. This pressure dependence is less evident with longer acid chain lengths: a pressure rise from 1 bar to 100 bar results in a 2% decrease in the dissociation constant of acetic acid but only a 0.8% decrease for butanoic acid. This pressure dependence is significantly smaller than raising the temperature of the bulk solution by 1 K, therefore, over the pressure range of this study, the effect of changes in the bulk dimerization constants due to pressure are neglected. In addition, although changes in dimerization may change the concentration axes of an adsorption isotherm, the adsorbed amount in the plateau region of the isotherm will not be affected.

Density variation with pressure:
The adsorption increase (as summarised in Fig. S1 below) with increasing pressure for acetic acid suggests an increase in the packing density of the adsorbed layers at the surface. In the bulk, the compression of a solution can be modelled with the isothermal compressibility coefficient, κ_p[33]

$$\frac{dv}{v} = -\kappa_p dP \tag{1}$$

Where v is the molar volume and P is the pressure. Therefore, the volume and density (ρ) can be written as:

$$v = v_0 e^{-\kappa_p (P - P_0)}$$
$$\rho = \rho_0 e^{\kappa_p (P - P_0)} \tag{2}$$

The density of pure acetic acid solution as a function of pressure at 2 temperatures (Vong et al[34]) along with the corresponding fits to equation (2) for density are presented in Fig. S1 where the bulk isotherm compressibility coefficient, κ_p, of acetic acid is calculated to be 8.4×10^{-5} bar^{-1}.

Similarly, the surface density data, from Table 3 in the paper, can also be considered with a similar density equation and enables us to estimate the surface adsorbed layer compressibility coefficient, κ_p, to be 6.1×10^{-3} bar^{-1}. The compressibility ratio of acetic acid in the bulk and

surface is significantly different with the 2D layer being approximately 2 orders of magnitude larger.

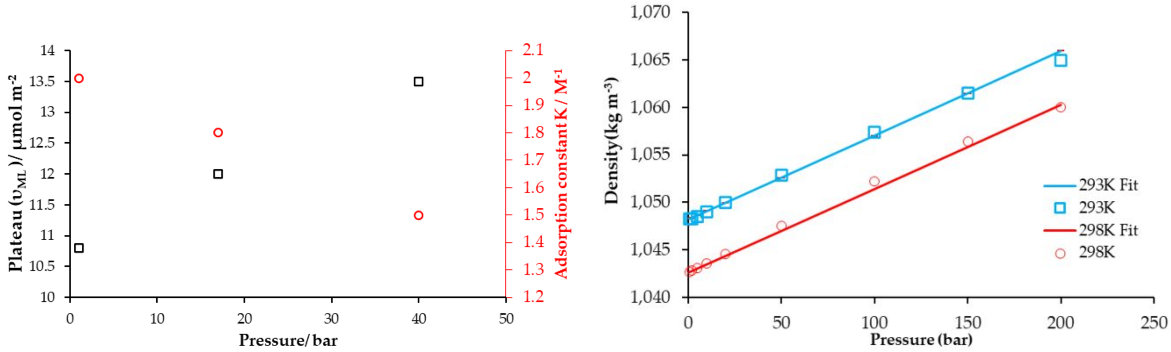

Figure S1. Left) The Langmuir constant and plateau adsorption for the adsorption isotherms of acetic acid in heptane as a function of pressure. See the text for the calculation of the surface adsorbed layer compressibility coefficient. Right) Bulk density data as a function of pressure as reported by Vong et al[34] and the corresponding fits to estimate the bulk compressibility coefficient, of acetic acid as 8.4×10^{-5} bar-1.

Partial Molar Volume of Mixing

Consider starting with pure species A and B, the volume of these species are given by:

$$\overline{V_{A,\,initial}} = X_A \overline{V_A^0}$$
$$\overline{V_{B,\,initial}} = X_B \overline{V_B^0}$$

The total initial volume is:

$$\overline{V_{tot,\ initial}} = X_A \overline{V_A^0} + X_B \overline{V_B^0}$$

Consider a ‘Regular solution’-type mixing where each molecule has its own volume for a mole of pure material, $\overline{V_A^0}$ and $\overline{V_B^0}$, but at any particular mixture concentration the partial molar volume depends on the concentration due to the non-ideal interaction. In the ‘regular solution’ type model the partial molar volume of each species can be written as:

$$\overline{V_A^R} = +\overline{V_A^0} + b_A {X_B}^2 \text{ and } \overline{V_B^R} = +\overline{V_B^0} + b_B {X_A}^2$$

Hence the total volume of a mixture with the composition of X_A of A and X_B of B is:

$$\overline{V_{mix}} = X_A \overline{V_A^R} + X_B \overline{V_B^R} = X_A\left(\overline{V_A^0} + b_A {X_B}^2\right) + X_B\left(+\overline{V_B^0} + b_B {X_A}^2\right)$$

Using the equation for $V_{tot,\ initial}$ above, the volume change on Mixing is:

$$\overline{\Delta V_{tot}} = \overline{V_{mix}} - \overline{V_{tot,\ initial}} = \left[X_A\left(\overline{V_A^0} + b_A {X_B}^2\right) + X_B\left(+\overline{V_B^0} + b_B {X_A}^2\right)\right] - \left[X_A \overline{V_A^0} + X_B \overline{V_B^0}\right]$$
$$\overline{\Delta V_{tot}} = \left[X_A\left(b_A {X_B}^2\right) + X_B\left(+b_B {X_A}^2\right)\right] = \left[X_A X_B (b_A X_B) + X_A X_B (+b_B X_A)\right]$$
$$\overline{\Delta V_{tot}} = X_A X_B [b_A X_B + b_B X_A]$$

From the Gibbs-Duhem equation[23], it can be shown that $b_A = b_B = b$, therefore:

$$\overline{\Delta V_{tot}} = X_A X_B b [X_B + X_A]$$

Using $X_B + X_A = 1$, the excess volume is given by:

$$\overline{\Delta V_{tot}} = X_A X_B b$$

This result is similar to the excess enthalpy in regular solution theory.

Stearic acid head group area calculation:

Data presented in the literature on the crystal structure of stearic acid[20] suggest stearic acid, C17H35COOH, has a monoclinic structure with a = 5.591 A, b = 7.404 A, c = 49.38 A and β= 117.22°. There are two molecules per unit cell, therefore, the area per head group is taken as a*b/2.
